# Heat stress induces expression of *HSP* genes in genetically divergent chickens

**DOI:** 10.1371/journal.pone.0186083

**Published:** 2017-10-11

**Authors:** Haniel Cedraz, Juliana Gracielle Gonzaga Gromboni, Antonio Amandio Pinto Garcia, Ronaldo Vasconcelos Farias Filho, Teillor Machado Souza, Eduardo Ribeiro de Oliveira, Elizangela Bonfim de Oliveira, Carlos Souza do Nascimento, Camila Meneghetti, Amauri Arias Wenceslau

**Affiliations:** 1 Universidade Estadual de Santa Cruz - UESC, Ilhéus, Bahia, Brazil; 2 Departament of Rural and Animal Technology - Universidade Estadual do Sudoeste da Bahia – Campus Itapetinga – UESB, Itapetinga, Bahia, Brazil; 3 Universidade Estadual de Santa Cruz - UESC, Ilhéus, Bahia, Brazil; 4 Department of Animal Science, Universidade Federal de Sergipe, São Cristovão, Sergipe, Brazil; University of PECS Medical School, HUNGARY

## Abstract

**Background:**

Chickens are animals that are sensitive to thermal stress, which may decrease their production level in terms that it affects feed intake and thus, decreasing body weight gain. The Heat Shock Factors (*HSF*) and Heat Shock Proteins (*HSP*) genes are involved in the key cellular defense mechanisms during exposure in hot environments. Aimed with this study to analyze the expression of *HSF1*, *HSF3*, *HSP70* and *HSP90* genes in two local breeds (Peloco and Caneluda) and a commercial broiler line (Cobb 500^®^) to verify differences in resistance of these chicken to Heat stress treatment. Chicken were submitted to heat stress under an average temperature of 39°C ± 1.

**Results:**

Under stress environment, the *HSP70* and *HSP90* genes were more expressed in backyard chickens than in broiler. There was a difference in *HSP70* and *HSP90* expression between Caneluda and Cobb and between Peloco and Cobb under stress and comfort environment respectively. HSP70 expression is higher in local breeds during heat stress than in a commercial broiler line. No significant differences were observed in the expression of *HSF1* and *HSF3* genes between breeds or environments.

**Conclusions:**

*HSP70* and *HSP90* genes are highly expressed, *HSF1* and *HSF3* genes did not have high expression in all genetic groups. *HSP70* and *HSP90* are highly expressed in Peloco and Caneluda within heat stress, these breeds proved to be very resistant to high temperature.

## Introduction

Poultry is one of the main sectors of the agribusiness producing thousands of tons of meat per year. However, environments with high temperatures may cause negative impacts on broiler’ physiology and production, leading to economic losses [1,2]. Broilers reached high levels of production due to genetic improvement, on the other hand, its metabolism become more accelerate, presenting poor thermoregulation, and as consequence being not well adapted to hot environments [3–5]. Differently, native backyard chickens are more adapted to environments in which they live, with rusticity that allow them to survive and reproduce constantly. These chicken are more resistant to high temperatures [6,7], however, they have low productive levels since they did not undergone genetic improvement and have low investment in breeding [8,9].

There are factors that act on defense mechanism against high temperatures. The ability of homeostasis can minimize extracellular damage [10] by altering gene expression in the presence of stress and returning to basal conditions after returning to thermal comfort conditions [11,12]. One of the defense mechanisms is the activation of more than 500 genes in the first ten minutes of exposure [13–15]. Among these genes are the *Heat Shock Factor* (HSF) leading the induction of gene expression [16,17] and the *Heat Shock Proteins* (HSP) that are some of the main defenses against heat stress [11,12].

The HSF1 and HSF3 are considered the main genes of HSF family in response to heat shock in chicken. Has been believed that induction of HSF1 and HSF3 in regulate HSP were bird-specific, however, a recent study has demonstrated that HSF1 and HSF3 have also regulate HSP70 expression in lizards and frogs [18]. *HSF1* is activated at low temperatures while *HSF3* continues to be activated at higher temperatures and longer exposure [19].The *HSP70* and *HSP90* genes are the most studied HSPs family and each has different functions [1]. The *HSP70* binds to newly synthesized proteins, preventing aggregation and assisting in folding [20,21], whereas *HSP90* interacts with proteins in older stages of folding, in addition to modifying the configuration of these proteins [22].

Many studies have reported genes related to heat stress in mammals [23,24], plants [25], fish [26] and broiler [27,28], but so far no studies have been identified the relation of chicken resistance to thermal stress with the expression of HSF's and HSP's genes. Aimed with this study to analyze the expression of *HSF1*, *HSF3*, *HSP70* and *HSP90* genes in two local breeds of chickens and a commercial chicken line in order to verify the resistance of these birds to heat stress.

## Material and methods

### Ethical approval

Experiment procedures were approved by the Ethics Committee on Animal Use—CEUA of Universidade Estadual do Sudoeste da Bahia (UESB), protocol 109/2015.

### Animals

In this study, we used 36 male and female chickens, being 12 chicks of each breed (Peloco and Caneluda (backyard breeds), and Cobb 500^®^ (commercial line)). Commercial birds were acquired a week after the birth of backyard chicken in the Universidade Estadual do Sudoeste da Bahia (UESB), Itapetinga, and raised under the same environmental conditions from November 2 to December 2 of 2015 with an average temperature of 26.5°C. The predominant climate of Itapetinga region is semi-arid, in which the temperature increases during the day and decrease during the night. Nutritional diet followed the requirements of the Brazilian Tables for Poultry and Swine [29] and the feed was produced in the poultry sector of UESB (Table 1). All chickens were raised in semi-open stalls and lined with wood shavings (wood chips).

**Table 1:** Initial feed used in the production of chicks up to 30 days of age (ROSTAGNO, GOMES, 2011).

Corn	61.1%
Soybean Meal	35.0%
Dicalcium Phosphate	2.00%
Limestone	1.10%
NaCl	0.30%
Vitamin And Mineral Supplement	0.40%
Nutritional Levels	
Crude Protein	21.2%
Metabolizable Energy	2.89%
Calcium	1.01%
Phosphor Available	0.49%
Sodium	1.63%
Lysine	1.10%
Methionine + Cysteine	0.74%

### Heat stress and collect of tissue samples

At 30 days of age all chicks were transferred to the climatic chambers. Birds were housed in groups of up to 12 chicks per cage. Heat stress was performed in two stages so that all chicken had the same slaughter age (30 days). First, six chicks of Peloco breed and six chicks of Caneluda breed were subjected to heat stress under an average temperature of 39.5°C and environmental relative humidity of 60% for 30 minutes. In the second stage, six chicks of Cobb 500^®^ line were subjected to heat stress with the same conditions of temperature, humidity and time. During the heat stress period, animals had *ad libitum* access to food and water.

During the heat stress, chickens were constantly observed for behavioral changes, in order to avoid deaths caused by excessive temperature. The acute heat stress was determined at the moment that most of the chicken (±90%) were prostrate (lying with the abdominal faced down), and with increased respiratory rate. Control chickens (six chicks of each genetic group) were slaughtered at the second stage of the experiment at 4 am (local time) to ensure thermal comfort temperature (23°C). All chicks (Heat stressed and comfort) were slaughtered by cervical dislocation.

After slaughter, samples of *Pectoralis major* muscle were collected, placed in cryogenic tubes, identified and stored in liquid nitrogen. Samples were transported to the Veterinary Genetics Laboratory at the Universidade Estadual de Santa Cruz (UESC), separated and stored in Ultrafreezer (-80°C).

### Extraction, quantification and quality of total RNA

For total RNA extraction, kit SV Total RNA Isolation System^®^ (Promega Corporation, Madison, USA) was used according to manufacturer's protocol. The concentration and quality of RNA were verified by NanoDrop 2000 spectrophotometer (Thermo Fisher Scientific Inc, Carlsbad, CA, USA) using the absorbance at 230, 260, 280nm. Besides, RNA integrity was analyzed by the presence of bands corresponding to 28S and 18S ribosomal RNAs observed through electrophoresis of 1 ug of RNA in 1% agarose gel stained with ethidium bromide.

### Reverse transcription of mRNA

The commercial kit GoScript TM Reverse Transcription System (Promega Corporation, Madison, USA) was used for reverse transcription of mRNA. Up to five micrograms of total RNA from samples were mixed to 1μl of Oligo(dT) (500μg/ml) and heated in 70°C for 5 minutes. After incubation, 4μl of 5X Reaction Buffer, 3.2μl MgCl_2_, 1μl dNTP (0,5mM), 1μl of reverse transcriptase enzyme, 0.5μl of inhibitor of recombinant ribonuclease RNaseOUT (20units) and ultrapure water completing 15μl. This mix were added to RNA+OligodT mix completing a volume total of 20μl and incubated on a thermocycler. Anneal at 25°C for 5 minutes; extend at 42°C for one hour, and 70°C for 15 minutes to inactivate the reverse transcriptase. After reverse transcription, cDNA was stored at -20°C. The concentration of cDNA was measured by NanoDrop 2000 spectrophotometer (Thermo Fisher Scientific Inc, Carlsbad, CA, USA) using the absorbance at 230, 260, 280nm.

### Target gene selection and optimization of RT-qPCR

Four target genes involved in regulation of heat stress in *Gallus gallus* were selected to be evaluated in different genetic groups (Table 2). To obtain the standard curve, we used a cDNA pool of all treatments aiming to optimize and calculate the PCR efficiency. We used three cDNA concentrations (15, 45 and 135ng/μl) and three primer concentrations (200, 400, 800 mM).

**Table 2:** Description of *G*. *gallus* genes related to heat stress, reference genes for chickens and their specific primers used in RT-qPCR analyzes. The primers of *HSF3*, *HSP70* and *HSP90* genes were designed by ALMEIDA, (2007).

GENE	DISCRIPTION	SEQUENCE (5'-3')	FUNCTION
HSF1*	Heat shock factor protein 1	F: TGTGGCTGATTCTTGGCTTT	Heat shock response
		R: GAGGGAGACAGAGGGGTTTC	
**HSF3**	Heat shock factor protein 3	F: CGGAAGATGGAAATGGAGAG	Heat shock response
		R: TCAGGAAGCAGGAGAGGAGA	
**HSP70**	Heat shock protein 70kDa	F: ATTCTTGCGTGGGTGTCTTC	Heat shock response
		R: GATGGTGTTGGTGGGGTTC	
**HSP90**	Heat shock protein 90kDa	F: TGAAACACTGAGGCAGAAGG	Heat shock response
		R: AAAGCCAGAGGACAGGAGAG	
MRPS27**	Mitochondrial ribosomal protein S27	F: GCTCCCAGCTCTATGGTTATG	Reference gene
		R: ATCACCTGCAAGGCTCTATTT	
RPL5**	Ribosomal protein L5	F: AATATAACGCCTGATGGGATGG	Reference gene
		R: CTTGACTTCTCTCTTGGGTTTCT	
MRPS30**	Mitochondrial ribosomal protein S30	F: CCTGAATCCCGAGGTTAACTATT	Reference gene
		R: GAGGTGCGGCTTATCATCTATC	

*Primer drawn by the authors of this work;

**Reference Genes obtained in previous studies [31].

RT-qPCR reaction conditions were set with initial denaturation temperature at 95°C for two minutes, and 40 cycles of denaturation at 95°C for 15 seconds. The extension temperature was individually standardized for each pair of primer for 60 seconds. At the end of amplification reaction, we included an additional step with gradual temperature increasing from 60 to 95°C for dissociation curve analysis. Amplification of all genes was performed in duplicate in a 7500 Fast Real Time PCR System (Applied Biosystems, Foster City, CA, USA). Results were obtained by using the Sequence Detection Systems software (V. 2.0.6) (Applied Biosystems Foster City, CA, USA) that generated the cycle threshold (Ct) parameter. The Ct values of duplicates were obtained directly from the above program and used to calculate the average Ct and standard deviation. PCR amplification efficiency was calculated for each reference gene using the following formula: E = (10^(-1/slope)-1^)x100 [30]. After efficiency analysis, the most appropriate annealing temperature and primer concentration were used in PCR reactions.

### Real time quantitative PCR

The reaction of RT-qPCR was performed using SYBR Green detection kit with GoTaq qPCR Master Mix (Promega, Madison, WI, EUA), using specific primers. Gene amplification was performed in duplicate using the Real Time PCR 7500 Fast system (Applied Biosystems, Foster City, CA, EUA) and results were obtained with the *Sequence Detection Systems* program (V. 2.0.6) (Applied Biosystems, Foster City, CA, EUA) that generated the *cycle threshold* (Ct) parameters.

The Ct values were exported to Microsoft Excel to calculate the Ct mean, standard deviation and the standard curve for each gene. A negative control (ultra-pure water) also was added in each assay. The qPCR reaction conditions were defined as follow: Initial denaturation at 95°C during ten minutes and 40 cycles of denaturation at 95°C for 15 seconds. The extension temperature between 60 and 64°C during one minute was ideal for all primers. Ct values of control wells were excluded from subsequent analyzes as well as the undetectable values.

### Statistical analysis of target genes

To perform the statistical analysis, %QPCR_MIXED [32] was used in the statistical software SAS^®^ 9.0. This macro performs analyzes by mixed linear models of RT-qPCR data. The program normalizes the data using the ΔΔCT method [33], thus generating *Fold Change*, which is the value of the relative expression between the control and the treatment [34].

In order to determine if there was difference between treatments (genetic groups and environment), contrasts were made between the factors comparing them to each other. Within this statistical model, the effects of genetic groups (Caneluda, Cobb and Peloco) and environment (comfort and thermal stress) were considered fixed, and the Genes factor was considered random. In this way it is possible to test the linear combinations between the levels of these factors (Comfort X Stress); (Cobb X Caneluda, Caneluda X Peloco and Cobb X Peloco) and also the variability between the genes in each treatment/breed, besides the interaction effects. Were considered different contrasts those that obtained p-value ≤ 0.05.

## Results

### Efficiency and specificity of primers

**Fig 1:**
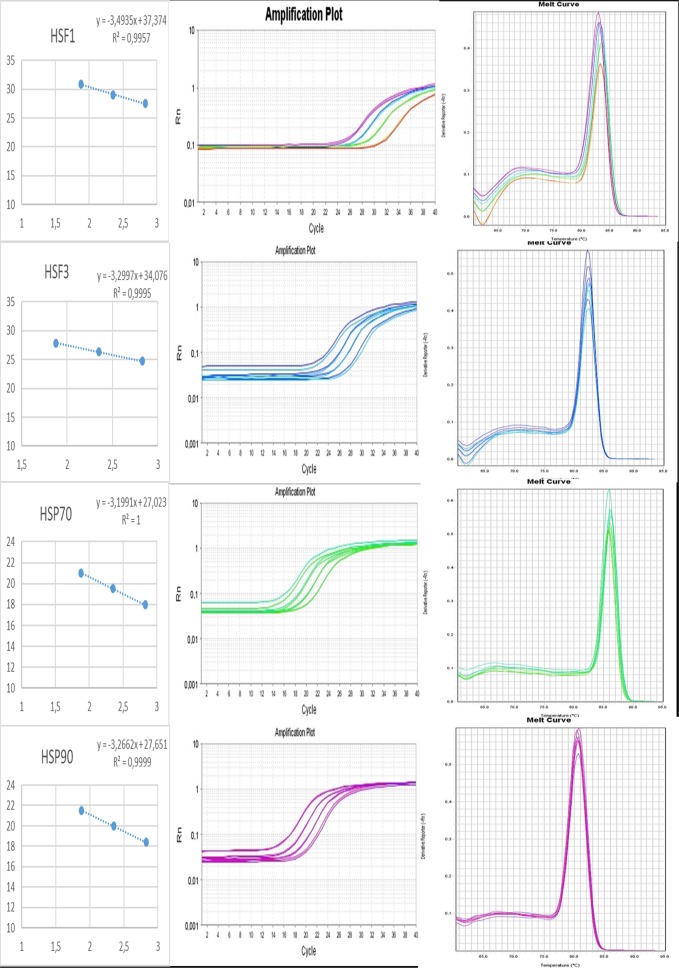
Regression curves, amplification and dissociation of the efficiency test for the 4 target genes (*HSF1*, *HSF3*, *HSP70* and *HSP90*) from broilers derived from RT-qPCR reactions. All dissociation curves show only one peak.

Prior to performing expression analysis of the interest genes, we performed efficiency test. The annealing temperature of 62°C was determined as optimal for all primers. The amplification efficiency varied between 93% and 105% corresponding to slope between -3.49 and -3.20. The coefficient of determination (R^2^) values were higher than 0.99 (Table 3). The primers specificity was evaluated through the dissociation curve, which showed only one peak indicating no primer dimers were detected and presenting excellent performance (Fig 1).

**Table 3:** Parameters of the specific primers of genes related to thermal stress and reference genes for broilers obtained from the analysis of efficiency curve in RT-qPCR.

GENE	AT (°C)	[CDNA]	[PRIMER]	EFFICIENCY (%)	R^2^	SLOPE
**HSF1**	62	45ng/μl	800mM	93	0.996	-3.494
**HSF3**	62	45ng/μl	800mM	101	0.999	-3.300
**HSP70**	62	45ng/μl	400mM	105	1	-3.199
**HSP90**	62	45ng/μl	400mM	102	0.999	-3.266
**MRPS27**	62	45ng/μl	800mM	105	0.998	-3.201
**RPL5**	62	45ng/μl	800mM	102	0.999	-3.284
**MRPS30**	62	45ng/μl	800mM	105	0.999	-3.207

AT = Annealing Temperature; *SLOPE* = Slope of the Line; R^2^ = Coefficient of Determination; [CDNA] = cDNA Concentration; [PRIMER] = Primer Concentration

### Descriptive statistics of target genes

Descriptive statistics were performed using BestKeeper tool [27]. It is possible to notice an expression variability through quantification cycles in four target genes, which was grouped into two categories (strong and moderate). Three genes (*HSP70*, *HSF1* and *HSP90*) had strong mRNA expression, with Ct values varying between 16 and 27 cycles, and one gene (*HSF3*) with moderate expression with 34 cycles [35] (Table 4).

**Table 4:** Descriptive statistics and expression levels of target genes related to heat stress in broilers (n = 36).

n = 36	HSF1	HSF3	HSP70	HSP90
geo Mean [Ct]	29.15	26.12	20.97	20.26
ar Mean [Ct]	29.19	26.16	21.16	20.33
min [Ct]	26.49	23.39	16.47	17.86
max [Ct]	33.92	30.25	26.35	24.96
**std dev [± Ct]**	**1.26**	**1.07**	**2.44**	**1.35**
**CV [% Ct]**	**4.33**	**4.08**	**11.54**	**6.66**
coeff. of corr. [r]	0.746	0.319	0.251	0.177

Abbreviations: [Ct] Cycle threshold; geo Mean [Ct]: Geometric mean of Ct; ar Mean [Ct]: Arithmetic mean of Ct; Min [Ct] and Max [Ct]: Ct threshold values; std dev [±Ct]: Standard deviation of Ct; CV [% Ct]: Coefficient of variation of Ct levels expressed as a percentage; SD and CV are indicated in bold.

### Relative expression of target genes

According to previous analysis [31], the MRPS27, RPL5 and MRPS30 genes were considered stable under genetic groups and environment factors which was performed a normalization factor by geometric mean. Therefore, these genes were used to normalize the relative expression of target genes.

**Fig 2:**
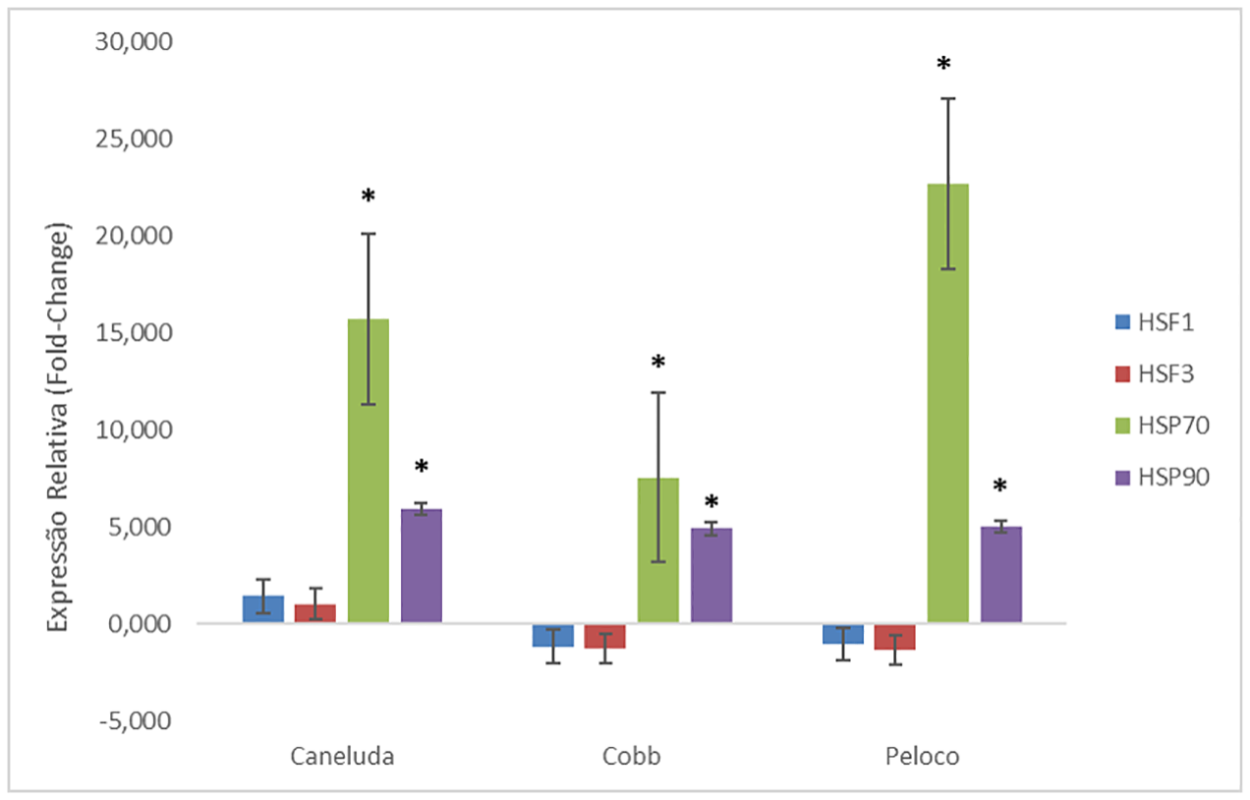
Relative expression analysis of *HSF1*, *HSF3*, *HSP70* and *HSP90* genes in different chicken genetic groups comparing comfort and thermal stress environments. *p-Value <0.05.

Comparing environments (stress X comfort), *HSF1* and *HSF3* genes were not significantly different among the three genetic groups. On the other hand, the *HSP70* genes had high expression and were statistically different within the three genetic groups (Table 5/Fig 2).

**Table 5:** Relative expression analysis of *HSF1*, *HSF3*, *HSP70* and *HSP90* genes in the different genetic groups of chicken comparing the comfort and thermal stress environments.

Gene	Comparison between treatment within genetic groups					
	Caneluda		Cobb		Peloco	
	Comfort X Stress		Comfort X Stress		Comfort X Stress	
	FC	*p-value*	FC	*p-value*	FC	*p-value*
**HSF1**	1.42	0.23	-1.17	0.58	-1.04	0.88
**HSF3**	1.03	0.90	-1.29	0.26	-1.35	0.19
**HSP70**	**15.71**	**<.0001**	**7.54**	**<.0001**	**22.67**	**<.0001**
**HSP90**	**5.92**	**<.0001**	**4.92**	**<.0001**	**5.01**	**<.0001**

**Fig 3:**
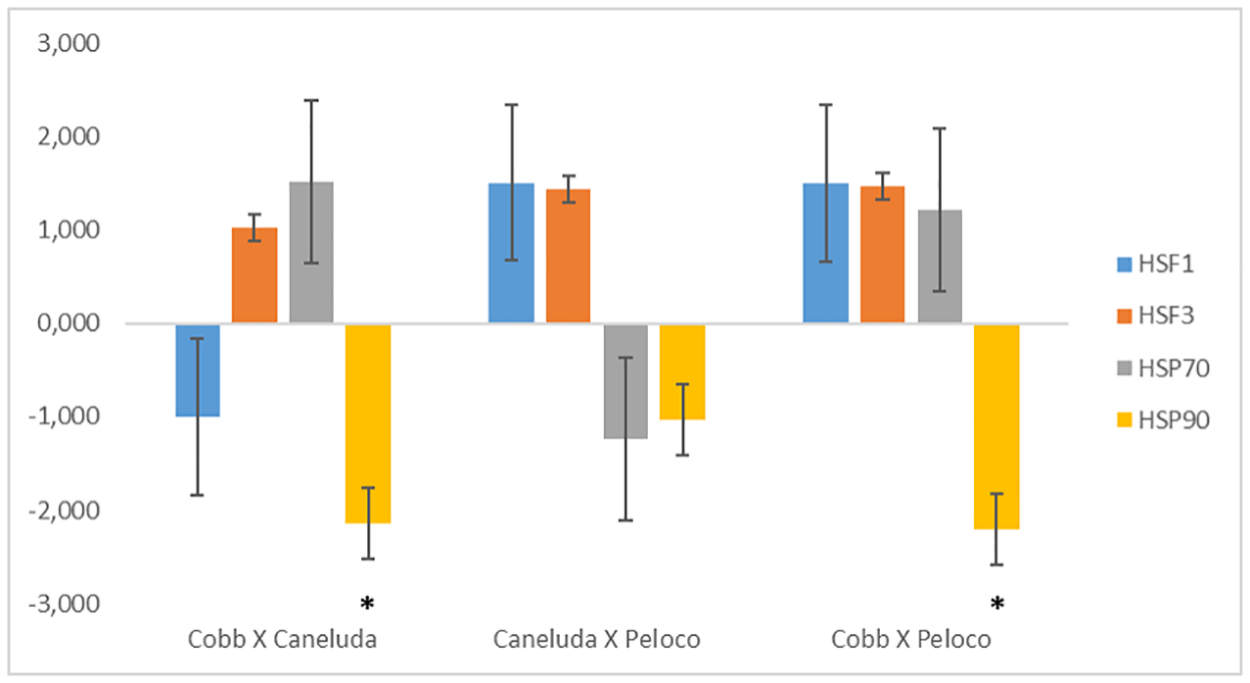
Relative expression analysis of *HSF1*, *HSF3*, *HSP70* and *HSP90* genes comparing the different chicken genetic groups within the thermal stress environment. *p-Value <0.05.

In thermal stress analysis, comparisons between genetic groups (Caneluda X Cobb, Caneluda X Peloco and Cobb X Peloco) were not significant for *HSF1*, *HSF3* and *HSP70* genes. In contrast, *HSP90* gene had a difference in relative expression in Cobb line compared to Caneluda and Peloco. In the comparison between Caneluda X Peloco, none of the analyzed genes showed a significant difference in relative expression (Table 6/Fig 3).

**Table 6:** Relative expression analysis of *HSF1*, *HSF3*, *HSP70* and *HSP90* genes comparing the different chicken genetic groups within the thermal stress environment.

Gene	Comparison between genetic groups under heat stress					
	Stress		Stress		Stress	
	Cobb X Caneluda		Caneluda X Peloco		Cobb X Peloco	
	FC	*p-value*	FC	*p-value*	FC	*p-value*
**HSF1**	-1.00	1.00	1.51	0.16	1.51	0.16
**HSF3**	1.02	0.92	1.44	0.11	1.47	0.09
**HSP70**	1.51	0.26	-1.24	0.56	1.22	0.58
**HSP90**	**-2.14**	**<.0001**	-1.03	0.89	**-2.20**	**<.0001**

**Fig 4:**
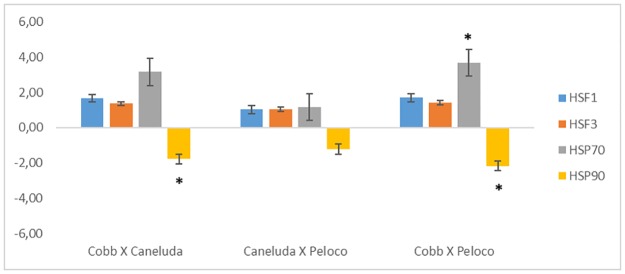
Relative expression analysis of *HSF1*, *HSF3*, *HSP70* and *HSP90* genes comparing the different genetic groups of chicken within the thermal comfort environment. *p-Value <0.05.

Breeds were also compared within thermal comfort. Only the *HSP70* and *HSP90* genes had statistically significant relative expression comparing Cobb X Caneluda and Cobb X Peloco. Comparing Caneluda X Peloco, none of the four genes showed difference in relative expression (Table 7/Fig 4).

**Table 7:** Relative expression analysis of *HSF1*, *HSF3*, *HSP70* and *HSP90* genes comparing the different chicken genetic groups within the thermal comfort environment.

Gene	Comparison between genetic groups under heat comfort					
	Comfort		Comfort		Comfort	
	Cobb X Caneluda		Caneluda X Peloco		Cobb X Peloco	
	FC	*p-value*	FC	*p-value*	FC	*p-value*
**HSF1**	1.65	0.08	1.02	0.94	1.69	0.07
**HSF3**	1.36	0.18	1.04	0.88	1.41	0.13
**HSP70**	**3.15**	**<.0001**	1.16	0.68	**3.67**	**<.0001**
**HSP90**	**-1.78**	**0.01**	-1.22	0.34	**-2.16**	**<.0001**

**Fig 5:**
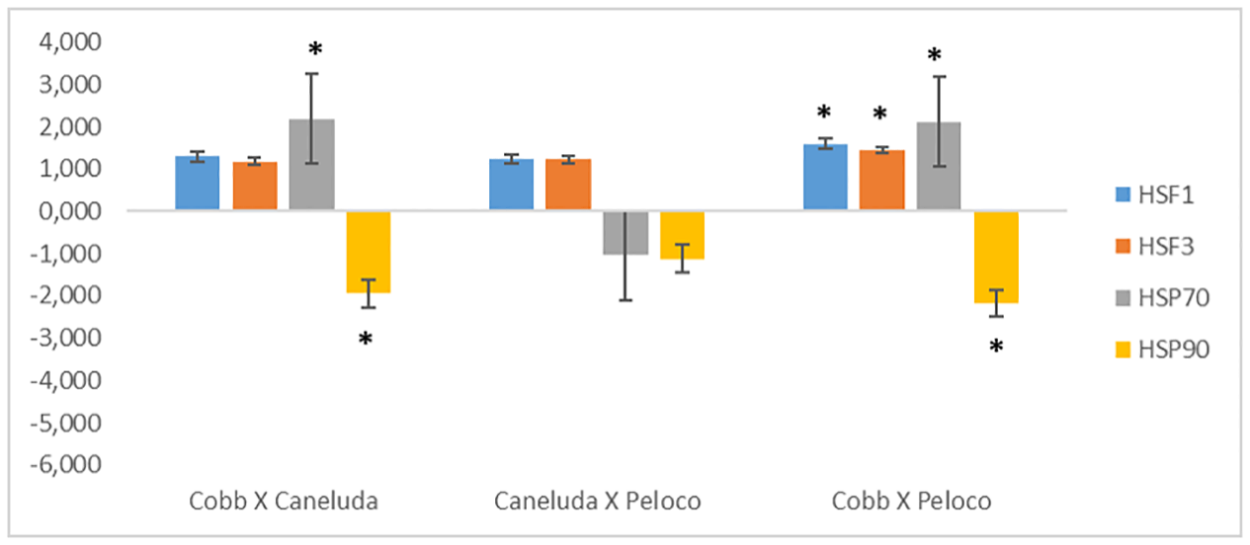
Relative expression analysis of *HSF1*, *HSF3*, *HSP70* and *HSP90* genes comparing the different genetic groups of chicken without considering the environments (comfort and thermal stress). *p-Value <0.05.

Comparing the genetic groups without considering environments, it was possible to notice that the commercial line Cobb 500^®^ had different relative expression in relation to the native breed Peloco (Peloco X Cobb) for all genes. While comparing Cobb 500^®^ to Caneluda (Caneluda X Cobb), there was only a significant difference for *HSP70* gene. In the comparison between Caneluda X Peloco, none of the four genes showed a significant difference in relative expression (Table 8/Fig 5).

**Table 8:** Relative expression analysis of HSF1, HSF3, HSP70 and HSP90 genes comparing the different genetic groups of chicken without considering the environments (comfort and thermal stress).

Gene	Comparison between genetic groups without considering the environments					
	Cobb X Caneluda		Caneluda X Peloco		Cobb X Peloco	
	FC	*p-value*	FC	*p-value*	FC	*p-value*
**HSF1**	1.29	0.22	1.24	0.29	**1.59**	**0.02**
**HSF3**	1.18	0.31	1.22	0.21	**1.44**	**0.02**
**HSP70**	**2.18**	**<.0001**	-1.03	0.90	**2.12**	**<.0001**
**HSP90**	**-1.95**	**<.0001**	-1.12	0.44	**-2.18**	**<.0001**

## Discussion

High temperatures can cause several damages to livestock production, especially in poultry farming causing financial losses. In addition to the technological mechanisms that try to alleviate the thermal stress in chicken, there are physiological factors that decrease the effects of heat. According to DE NADAL et al., (2011) [10] exposure to thermal stress can promote expression of genes related to survival while not expressing less essential genes, resulting in the rapid expression of Heat Shock Factors (HSF) and Heat Shock Protein (HSP) [36,37]

In this study, *HSF1* and *HSF3* genes showed low relative expression in all treatments (heat stress and thermal comfort), and had a difference in expression for Cobb compared to Peloco. These genes are not well expressed in acute thermal stress. *HSF1* is activated in medium heat stress whereas *HSF3* is activated in chronic thermal stress [19,37]. This suggests that *HSF3* may play an important role in long periods of heat stress in chicken [36].

Some studies in humans [38] and plants [39–41] have shown that different types of stress can promote the HSF family genes expression. The response of HSF genes during thermal stress may be involved in expression of HSP's genes [1,42], however, this mechanism is not yet well known [43]. *HSF1* induces only *HSP70* [44] whereas *HSF3* promotes the expression of all HSPs in chicken [45]. In addition, PRAKASAM et al. (2013) [46] have demonstrated that *HSF3* is also involved in the expression of IL-6 pyrogenic cytokine during thermal stress.

Heat shock proteins produce responses to temperature rise and are driven by some factors besides heat, such as microbial infection, tissue trauma and genetic injury [47]. In this study, *HSP70* and *HSP90* genes had a significant difference in relative expression in all comparisons, especially while comparing native chicken to commercial line Cobb, since the last one is more sensitive to heat stress [48–52].

In comparison between thermal comfort and heat stress, Caneluda and Peloco had high expression of *HSP70* while Cobb had medium relative expression in a thermal stress environment. Even with high expression of *HSP70* gene, the local breeds remained comfortable during the thermal stress, while commercial line chicken showed a great level of discomfort, suggesting that the genes played a protective role. The *HSP90* gene had medium expression in the three genetic groups. In heat environments, *HSP70* gene expression plays a better role in cellular functions than *HSP90* [47].

Within the thermal comfort environment, Cobb chicken had higher expression of *HSP70* and *HSP90* than Caneluda and Peloco, even though the expression of these genes has been low. As commercial chicken are genetically improved for production traits [3], the maintenance characteristics are decreased and these chickens are not adapted to warm environment conditions [8]. In this way, breeding environment could influence the expression of these genes in terms that in production environment the temperature was high and not controlled. Besides that, natural selection has been acting in Peloco and Caneluda chickens making them resistance to high temperatures from tropical weather, which seems have a negative correlation between production traits and heat resistance.

In relation to heat stress environment, only *HSP90* gene had significant expression in Caneluda animals compared to Cobb (p-value = 0.0004) and in Peloco chicken also compared to Cobb line (p-value = 0.0003). Comparing Caneluda and Peloco, there was no significant difference in *HSP90* expression. Caneluda and Peloco are extensively reared animals, being more adapted to the warm environment and able to stay under thermal stress easily than Cobb, therefore, these wild chicken are more resistant to heat, even at temperatures higher than they are used to.

In gene expression analysis without considering heat stress and thermal comfort, there was difference in expression only in the comparison between Cobb and Peloco for all evaluated genes. The *HSF1*, *HSF3* and *HSP70* genes were more expressed in Peloco, while *HSP90* was more expressed in Cobb. In comparison between Caneluda X Cobb there was no significant difference in expression of HSF's genes, however, the *HSP70* gene was more expressed in Caneluda and the *HSP90* more expressed in Cobb.

Some studies have shown changes in HSP expression from heart, liver, kidney, blood and muscle of broilers [1,53,54]. The results of *HSP70* and *HSP90* genes presented in this study are in agreement with those reported by LEI et al. (2009) [54], XIE et al., (2014)[1] and YU et al., (2008)[53]. *HSF1* and *HSF3* genes showed different results than those observed by XIE et al., (2014) [1], which reported high expression of these genes in chickens submitted to thermal stress. This inconsistency of results may have been due to the induction method at high temperatures and the time of exposure of the animals.

It is important to have more studies using these genetic groups to construct a molecular profile in relation to thermal stress, using these and other genes of the HSF's and HSP's family, besides genes that are directly and indirectly related to thermal stress in chickens.

## Conclusion

Given the above, it can be stated that *HSP70* and *HSP90* genes are highly expressed in all evaluated genetic groups. The *HSF1* and *HSF3* genes did not have high expression in the studied genetic groups neither in comfort and stress environments, whereas *HSP70* and *HSP90* are highly expressed in Peloco and Caneluda within thermal stress, these breeds proved to be very resistant to high temperature.

## Supporting information

S1 Table Data of target gene expression. The file contains raw data of Target Genes expression as Ct. 32 samples, two factors and four target genes.
(XLSX)

## References

[1] Xie J, Tang L, Lu L, Zhang L, Xi L, Liu H-C, et al Differential Expression of Heat Shock Transcription Factors and Heat Shock Proteins after Acute and Chronic Heat Stress in Laying Chickens (Gallus gallus). Cotterill S, editor. PLoS One [Internet]. 2014;9:e102204 Available from: http://dx.plos.org/10.1371/journal.pone.0102204 25072282 10.1371/journal.pone.0102204 PMC4114549

[2] Temim S, Chagneau a M, Peresson R, Tesseraud S. Chronic heat exposure alters protein turnover of three different skeletal muscles in finishing broiler chickens fed 20 or 25% protein diets. J. Nutr. [Internet]. 2000;130:813–9. Available from: http://www.ncbi.nlm.nih.gov/pubmed/10736335 10736335 10.1093/jn/130.4.813

[3] Mariante AS, Albuquerque MSM, Egito AA, Mcmanus C. Present status of the conservation of livestock genetic resources in Brazil ☆. Livest. Sci. [Internet]. Elsevier B.V.; 2009;120:204–12. Available from: 10.1016/j.livsci.2008.07.007

[4] CLASSEN HL. Managing metabolic disease in rapidly growing strains of poultry In: HILL WG, BISHOP SC, MCGUIRK B, MCKAY JC, SIMM G, WEBB A, editors. Chall. Genet. Chang. Anim. Prod. Publicatio. Edinburgh: Journal of the British Society of Animal Science; 2000 p. 63–4.

[5] Federal U, Rio DO, Do G, Agronomia FDE. Otimização da produção de frango de corte em condições de estresse por calor. 2005;

[6] Duangjinda M, Tunim S, Duangdaen C, Boonkum W. Hsp70 Genotypes and Heat Tolerance of Commercial and Native Chickens Reared in Hot and Humid Conditions. Brazilian J. Poult. Sci. 2017;19:7–18.

[7] Soleimani AF, Zulkifli I, Omar AR, Raha AR. Physiological responses of 3 chicken breeds to acute heat stress. Poult. Sci. [Internet]. 2011;90:1435–40. Available from: https://academic.oup.com/ps/article-lookup/doi/10.3382/ps.2011-01381 21673158 10.3382/ps.2011-01381

[8] ALMEIDA ECDJ. DIVERSIDADE FENOTÍPICA DE GALINHAS NATIVAS DA RAÇA PELOCO COM BASE EM DESCRITORES FENOTÍPICOS SOB ANÁLISE MULTIVARIADA. Universidade Estadual do Sudoeste da Bahia; 2013.

[9] Edvaldo Sagrilo, Girão ES, Barbosa FJV, Ramos GM, Azevedo JN de, Medeiros LP, et al. Agricultura Familiar—Galinha Caipira [Internet]. Ministério do Desenvolv. Soc. e Combat. à Fome. 2003 [cited 2016 Aug 6]. p. 1–2. http://www.mds.gov.br/falemds/perguntas-frequentes/bolsa-familia/programas-complementares/beneficiario/agricultura-familiar

[10] de Nadal E, Ammerer G, Posas F. Controlling gene expression in response to stress. Nat. Rev. Genet. [Internet]. Nature Publishing Group; 2011;12:833–45. Available from: 10.1038/nrg3055 22048664

[11] Gasch a P, Spellman PT, Kao CM, Carmel-Harel O, Eisen MB, Storz G, et al Genomic expression programs in the response of yeast cells to environmental changes. Mol. Biol. Cell. 2000;11:4241–57. 11102521 10.1091/mbc.11.12.4241 PMC15070

[12] Causton HC, Ren B, Koh SS, Harbison CT, Kanin E, Jennings EG, et al Remodeling of yeast genome expression in response to environmental changes. Mol Biol Cell [Internet]. 2001;12:323–37. Available from: http://www.ncbi.nlm.nih.gov/entrez/query.fcgi?cmd=Retrieve&db=PubMed&dopt=Citation&list_uids=11179418 11179418 10.1091/mbc.12.2.323 PMC30946

[13] Capaldi AP, Kaplan T, Liu Y, Habib N, Regev A, Friedman N, et al Structure and function of a transcriptional network activated by the MAPK Hog1. Nat. Genet. [Internet]. 2008;40:1300–6. Available from: 10.1038/ng.235 18931682 PMC2825711

[14] Ni L, Bruce C, Hart C, Leigh-Bell J, Gelperin D, Umansky L, et al Dynamic and complex transcription factor binding during an inducible response in yeast. Genes Dev. 2009;23:1351–63. 10.1101/gad.1781909 19487574 PMC2701586

[15] Miller C, Schwalb B, Maier K, Schulz D, Dümcke S, Zacher B, et al Dynamic transcriptome analysis measures rates of mRNA synthesis and decay in yeast. Mol. Syst. Biol. 2011;7:458 10.1038/msb.2010.112 21206491 PMC3049410

[16] Guertin MJ, Petesch SJ, Zobeck KL, Min IM, Lis JT. Drosophila heat shock system as a general model to investigate transcriptional regulation. Cold Spring Harb. Symp. Quant. Biol. [Internet]. 2010;75:1–9. Available from: http://www.ncbi.nlm.nih.gov/pubmed/21467139%5Cnhttp://www.ncbi.nlm.nih.gov/entrez/query.fcgi?cmd=Retrieve&db=PubMed&dopt=Citation&list_uids=21467139 10.1101/sqb.2010.75.039 21467139 PMC5967404

[17] Weake VM, Workman JL. Inducible gene expression: diverse regulatory mechanisms. Nat. Rev. Genet. [Internet]. 2010;11:426–37. Available from: http://www.ncbi.nlm.nih.gov/entrez/query.fcgi?db=pubmed&cmd=Retrieve&dopt=AbstractPlus&list_uids=20421872 10.1038/nrg2781 20421872

[18] Takii R, Fujimoto M, Matsuura Y, Wu F, Oshibe N, Takaki E, et al HSF1 and HSF3 cooperatively regulate the heat shock response in lizards. PLoS One. 2017;12:1–20. 10.1371/journal.pone.0180776 PMC5501597 28686674

[19] Tanabe M, Nakai A, Kawazoe Y, Nagata K. Different thresholds in the responses of two heat shock transcription factors, HSF1 and HSF3. J. Biol. Chem. 1997;272:15389–95. 9182569 10.1074/jbc.272.24.15389

[20] Hartl FU. Molecular Chaperones in the Cytosol: from Nascent Chain to Folded Protein. Science (80-.). [Internet]. 2002;295:1852–8. Available from: http://www.sciencemag.org/cgi/doi/10.1126/science.1068408 10.1126/science.1068408 11884745

[21] Hartl F-U, Hlodan R, Langer T. Molecular chaperones in protein folding: the art of avoiding sticky situations. Trends Biochem. Sci. [Internet]. 1994;19:20–5. Available from: http://linkinghub.elsevier.com/retrieve/pii/0968000494901694 7908149 10.1016/0968-0004(94)90169-4

[22] Wegele H, Müller L, Buchner J. Hsp70 and Hsp90—a relay team for protein folding. Rev. Physiol. Biochem. Pharmacol. 2004;151:1–44. 10.1007/s10254-003-0021-1 14740253

[23] Sonna LA, Fujita J, Gaffin SL, Lilly CM. Invited review: Effects of heat and cold stress on mammalian gene expression. J. Appl. Physiol. [Internet]. 2002 [cited 2016 Jun 19];92:1725–42. Available from: http://www.ncbi.nlm.nih.gov/pubmed/11896043 10.1152/japplphysiol.01143.2001 11896043

[24] Ahn S-G, Thiele DJ. Redox regulation of mammalian heat shock factor 1 is essential for Hsp gene activation and protection from stress. Genes Dev. [Internet]. 2003 [cited 2016 Jun 19];17:516–28. Available from: http://www.ncbi.nlm.nih.gov/pubmed/12600944 10.1101/gad.1044503 12600944 PMC195992

[25] Nagaya S, Kawamura K, Shinmyo A, Kato K. The HSP terminator of arabidopsis thaliana increases gene expression in plant cells. Plant Cell Physiol. 2010;51:328–32. 10.1093/pcp/pcp188 20040586

[26] Yeh FL, Hsu T. Differential regulation of spontaneous and heat-induced HSP 70 expression in developing zebrafish (Danio rerio). J. Exp. Zool. 2002;293:349–59. 10.1002/jez.10093 12210118

[27] Figueiredo D, Gertler A, Cabello G, Decuypere E, Buyse J, Dridi S. Leptin downregulates heat shock protein-70 (HSP-70) gene expression in chicken liver and hypothalamus. Cell Tissue Res. 2007;329:91–101. 10.1007/s00441-007-0414-6 17406896

[28] Lei L, Hepeng L, Xianlei L, Hongchao J, Hai L, Sheikhahmadi A, et al Effects of acute heat stress on gene expression of brain-gut neuropeptides in broiler chickens (Gallus gallus domesticus). J. Anim. Sci. [Internet]. 2014;91:5194–201. Available from: http://www.animalsciencepublications.org/publications/jas/abstracts/91/11/5194 10.2527/jas.2013-6538 23989874

[29] Rostagno HS, Gomes PC. Composição de Alimentos e Exigências Nutricionais 3 a Edição Editor : Horacio Santiago Rostagno. 2011;

[30] Pfaffl MW. A new mathematical model for relative quantification in real-time RT-PCR. Nucleic Acids Res. [Internet]. 2001;29:e45 Available from: http://www.ncbi.nlm.nih.gov/pubmed/11328886 11328886 10.1093/nar/29.9.e45 PMC55695

[31] Cedraz de Oliveira H, Pinto Garcia AA, Gonzaga Gromboni JG, Vasconcelos Farias Filho R, Souza do Nascimento C, Arias Wenceslau A. Influence of heat stress, sex and genetic groups on reference genes stability in muscle tissue of chicken. Schönbach C, editor. PLoS One [Internet]. R Foundation for Statistical Computing; 2017 [cited 2017 May 9];12:e0176402 Available from: http://dx.plos.org/10.1371/journal.pone.0176402 10.1371/journal.pone.0176402 PMC5411030 28459824

[32] Steibel JP, Poletto R, Coussens PM, Rosa GJM. A powerful and flexible linear mixed model framework for the analysis of relative quantification RT-PCR data. Genomics [Internet]. 2009;94:146–52. Available from: http://linkinghub.elsevier.com/retrieve/pii/S0888754309000986 10.1016/j.ygeno.2009.04.008 19422910

[33] Livak KJ, Schmittgen TD. Analysis of relative gene expression data using real-time quantitative PCR and. Methods. 2001;25:402–8. 10.1006/meth.2001.1262 11846609

[34] Fu WJ, Hu J, Spencer T, Carroll R, Wu G. Statistical models in assessing fold change of gene expression in real-time RT-PCR experiments. Comput. Biol. Chem. 2006;30:21–6. 10.1016/j.compbiolchem.2005.10.005 16321570

[35] Porterfield A. What Is a Ct Value? [Internet]. 2015 [cited 2017 Feb 23]. http://bitesizebio.com/24581/what-is-a-ct-value/

[36] Pardue ML, Ballinger DG, Hogan NC. The heat shock response. Cells coping with transient stress. Ann. N. Y. Acad. Sci. [Internet]. 1992;663:125–38. Available from: http://www.ncbi.nlm.nih.gov/pubmed/1482046 1482046 10.1111/j.1749-6632.1992.tb38656.x

[37] Morimoto R. Cells in Stress: Transcriptional Activation of Heat Shock Genes. Science (80-.). [Internet]. 1993;259:1409–10. Available from: http://www.biochem.northwestern.edu/ibis/morimoto/research/Publications/MorimotoScience93.pdf 10.1126/science.8451637 8451637

[38] Ding XZ, Smallridge RC, Galloway RJ, Kiang JG. Rapid assay of HSF1 and HSF2 gene expression by RT-PCR. Mol. Cell. Biochem. 1987;158:189–92. 10.1007/BF00225845 8817481

[39] Hu W, Hu G, Han B. Genome-wide survey and expression profiling of heat shock proteins and heat shock factors revealed overlapped and stress specific response under abiotic stresses in rice. Plant Sci. 2009;176:583–90. 10.1016/j.plantsci.2009.01.016 26493149

[40] Mittal D, Chakrabarti S, Sarkar A, Singh A, Grover A. Heat shock factor gene family in rice: Genomic organization and transcript expression profiling in response to high temperature, low temperature and oxidative stresses. Plant Physiol. Biochem. [Internet]. Elsevier Masson SAS; 2009;47:785–95. Available from: 10.1016/j.plaphy.2009.05.003 19539489

[41] Swindell WR, Huebner M, Weber AP. Transcriptional profiling of Arabidopsis heat shock proteins and transcription factors reveals extensive overlap between heat and non-heat stress response pathways. BMC Genomics [Internet]. 2007;8:125 Available from: http://www.pubmedcentral.nih.gov/articlerender.fcgi?artid=1887538&tool=pmcentrez&rendertype=abstract 10.1186/1471-2164-8-125 17519032 PMC1887538

[42] Nakai A, Ishikawa T. Cell cycle transition under stress conditions controlled by vertebrate heat shock factors. EMBO J. 2001;20:2885–95. 10.1093/emboj/20.11.2885 11387221 PMC125499

[43] Xue H, Slavov D, Wischmeyer PE. Glutamine-mediated dual regulation of heat shock transcription factor-1 activation and expression. J. Biol. Chem. 2012;287:40400–13. 10.1074/jbc.M112.410712 23055521 PMC3504755

[44] Inouye S, Katsuki K, Izu H, Fujimoto M, Sugahara K, Yamada S, et al Activation of heat shock genes is not necessary for protection by heat shock transcription factor 1 against cell death due to a single exposure to high temperatures. Mol Cell Biol [Internet]. 2003;23:5882–95. Available from: http://www.ncbi.nlm.nih.gov/pubmed/12897157 10.1128/MCB.23.16.5882-5895.2003 12897157 PMC166333

[45] Tanabe M, Kawazoe Y, Takeda S, Morimoto RI, Nagata K, Nakai A. Disruption of the HSF3 gene results in the severe reduction of heat shock gene expression and loss of thermotolerance. EMBO J. 1998;17:1750–8. 10.1093/emboj/17.6.1750 9501096 PMC1170522

[46] Prakasam R, Fujimoto M, Takii R, Hayashida N, Takaki E, Tan K, et al Chicken IL-6 is a heat-shock gene. FEBS Lett. [Internet]. Federation of European Biochemical Societies; 2013;587:3541–7. Available from: 10.1016/j.febslet.2013.09.012 24055475

[47] Ali N, Banu N. Heat Shock Proteins: Molecular Chaperones. Aligarh Dep. Biochem. 1991;19:166–72.

[48] Mello JLM, Boiago MM, Giampietro-Ganeco A, Berton MP, Vieira LDC, Souza RA, et al Periods of heat stress during the growing affects negatively the performance and carcass yield of broilers. Arch. Zootec. 2015;64:339–45.

[49] Altan Ö, Pabuçcuoğlu A, Altan A, Konyalioğlu S, Bayraktar H. Effect of heat stress on oxidative stress, lipid peroxidation and some stress parameters in broilers. Br. Poult. Sci. [Internet]. 2003;44:545–50. Available from: http://www.tandfonline.com/doi/abs/10.1080/00071660310001618334 10.1080/00071660310001618334 14584844

[50] Akbarian A, Michiels J, Degroote J, Majdeddin M, Golian A, De Smet S. Association between heat stress and oxidative stress in poultry; mitochondrial dysfunction and dietary interventions with phytochemicals. J. Anim. Sci. Biotechnol. [Internet]. 2016;7:37 Available from: http://jasbsci.biomedcentral.com/articles/10.1186/s40104-016-0097-5 10.1186/s40104-016-0097-5 27354915 PMC4924307

[51] Al-Batshan HA. Performance and Heat Tolerance of Broilers as Affected by Genotype and High Ambient Temperature. Asian-Australasian J. Anim. Sci. 2002;15:1502–6.

[52] Lara JL, Rostagno HM. Impact of Heat Stress on Poultry Production. Animals. 2013. 10.3390/ani3020356 PMC4494392 26487407

[53] Yu J, Bao E, Yan J, Lei L. Expression and localization of Hsps in the heart and blood vessel of heat-stressed broilers. Cell Stress Chaperones. 2008;13:327–35. 10.1007/s12192-008-0031-7 18350374 PMC2673943

[54] Lei L, Yu J, Bao E. Expression of heat shock protein 90 (Hsp90) and transcription of its corresponding mRNA in broilers exposed to high temperature. Br. Poult. Sci. [Internet]. 2009;50:504–11. Available from: http://www.ncbi.nlm.nih.gov/pubmed/19735020 10.1080/00071660903110851 19735020

